# RETRACTION: A Meta‐Analysis Comparing Efficiency of Limb‐Salvage Surgery vs Amputation on Patients With Osteosarcoma Treated With Neoadjuvant Chemotherapy

**DOI:** 10.1111/iwj.70339

**Published:** 2025-03-17

## Abstract

**RETRACTION:** AbdelgawadM. A. , ParambiD. G. T. , GhoneimM. M. , AlotaibiN. H. , AlzareaA. I. , HassanA. H. M. , and AbdelrahimM. E. A. , “A Meta‐Analysis Comparing Efficiency of Limb‐Salvage Surgery vs Amputation on Patients With Osteosarcoma Treated With Neoadjuvant Chemotherapy,” International Wound Journal 19, no. 7 (2022): 1616–1624, 10.1111/iwj.13758.35122396 PMC9615273

The above article, published online on 05 February 2022, in Wiley Online Library (http://onlinelibrary.wiley.com/), has been retracted by agreement between the journal Editor in Chief, Professor Keith Harding; and John Wiley & Sons Ltd. Following an investigation by the publisher, all parties have concluded that this article was accepted solely on the basis of a compromised peer review process. The editors have therefore decided to retract the article. The authors did not indicate their agreement with the retraction.

**RETRACTION:** M. A. Abdelgawad , D. G. T. Parambi , M. M. Ghoneim , N. H. Alotaibi , A. I. Alzarea , A. H. M. Hassan , and M. E. A. Abdelrahim , “A Meta‐Analysis Comparing Efficiency of Limb‐Salvage Surgery vs Amputation on Patients With Osteosarcoma Treated With Neoadjuvant Chemotherapy,” International Wound Journal 19, no. 7 (2022): 1616–1624, 10.1111/iwj.13758.35122396 PMC9615273

The above article, published online on 05 February 2022, in Wiley Online Library (http://onlinelibrary.wiley.com/), has been retracted by agreement between the journal Editor in Chief, Professor Keith Harding; and John Wiley & Sons Ltd. Following an investigation by the publisher, all parties have concluded that this article was accepted solely on the basis of a compromised peer review process. The editors have therefore decided to retract the article. The authors did not indicate their agreement with the retraction.

